# Vonoprazan-Based Regimen Is More Useful than PPI-Based One as a First-Line* Helicobacter pylori* Eradication: A Randomized Controlled Trial

**DOI:** 10.1155/2017/4385161

**Published:** 2017-02-28

**Authors:** Masafumi Maruyama, Naoki Tanaka, Daisuke Kubota, Masayuki Miyajima, Takefumi Kimura, Koujiro Tokutake, Ryujiro Imai, Toru Fujisawa, Hiromitsu Mori, Yoshiaki Matsuda, Shuichi Wada, Akira Horiuchi, Kendo Kiyosawa

**Affiliations:** ^1^Department of Gastroenterology, Nagano Red Cross Hospital, Nagano, Japan; ^2^Department of Metabolic Regulation, Shinshu University Graduate School of Medicine, Matsumoto, Japan; ^3^Digestive Disease Center, Showa Inan General Hospital, Komagane, Japan; ^4^Department of Gastroenterology, Aizawa Hospital, Matsumoto, Japan

## Abstract

*Background*. A new agent, potassium-competitive acid blocker vonoprazan (VPZ) has potent acid-inhibitory effects and may offer advantages over conventional* H. pylori* eradication therapies. We aimed to compare the eradication rate between VPZ-based treatment and PPI-based one.* Methods*. This randomized controlled trial was designed to assign 141 patients with* H. pylori-*positive gastritis to VPZ group (VPZ 20 mg, amoxicillin 750 mg, and clarithromycin 200 or 400 mg twice daily for 7 days) or PPI group (rabeprazole 20 mg or lansoprazole 30 mg, amoxicillin 750 mg, and clarithromycin 200 or 400 mg twice daily for 7 days). Primary endpoints were eradication rates and adverse events.* Results*. Seventy of 72 patients in VPZ group and 63 of 69 patients in PPI group completed the treatment after 7 days. The eradication rate was significantly higher in VPZ group than PPI group by intention-to-treat analysis (95.8% versus 69.6%, *P* = 0.00003, 95% confidence interval [CI] 88.3-99.1% versus 57.3-80.1%) and per-protocol analysis (95.7% versus 71.4%, *P* = 0.0002, 95% CI 88.0-99.1% versus 58.7-82.1%). The incidence of adverse events was not different between the groups (26.3% in VPZ group versus 37.7% in PPI group, *P* = 0.15).* Conclusion*. VPZ-based regimen is more useful than that PPI-based regimen as a first-line* H. pylori* eradication therapy.

## 1. Introduction

Helicobacter pylori (*H. pylori*) is a main cause of chronic gastritis and is related to gastric carcinogenesis [1]. A large-scale prospective randomized study clearly demonstrated that* H. pylori* eradication therapy deduced the relative risk of gastric cancer [2, 3]. Since* H. pylori* serves as an initiator and promotor during gastric carcinogenic process [4–6], it is widely accepted that complete eradication of* H. pylori* is beneficial for chronic gastritis patients.

The most widely prescribed regimen for a first-line* H. pylori* eradication has been a triple therapy with proton pump inhibitor (PPI), amoxicillin (AMX), and clarithromycin (CLR) [7–10]. However, the eradication rate of PPI-based regimen has been declining over the years, mainly due to insufficient effects to antibiotics [11–14]. To exert sufficient* H. pylori*-killing effect of AMX and CLR, maintenance of intragastric pH around 6 to 7 is mandatory [15]. Therefore, development of potent gastric acid secretion-lowering agents is desired to improve the eradication rate.

Vonoprazan (VPZ), a first-in-class potassium-competitive blocker, can inhibit the H^+^, K^+^-ATPase-mediated gastric acid secretion in a reversible and potassium-competitive manner, possessing approximately 350 times more potent inhibitory effects than lansoprazole (LPZ), a typical PPI, in in vitro experiments [16–18]. VPZ is very stable in gastric juice, works immediately, and the effect lasts for a long time. Additionally, it undergoes substantial metabolic elimination independently of cytochrome P450 (CYP) 2C19 polymorphism [19, 20], which is distinct from PPI. This new agent is expected to offer clinical advantages over conventional PPI-based eradication therapies.

Recently, Murakami et al. reported the results of phase III noninferior trial that VPZ/AMX/CLR combination therapy is effective as a first- and second-line eradication for* H. pylori*-positive patients with a history of gastric or duodenal ulcer [21]. However, there is no prospective study verifying the superiority of VPZ-based regimen for a first-line* H. pylori* eradication. Therefore, we designed this randomized trial in order to compare the efficacy and safety between VPZ-based therapy and conventional PPI-based one.

## 2. Materials and Methods

### 2.1. Ethics

This study was conducted in accordance with the Declaration of Helsinki. The study protocol was approved on the Institutional Review Board of Nagano Red Cross Hospital and was registered in University Hospital Medical Information Network (registration number: ID21148) [http://www.umin.ac.jp/].

### 2.2. Study Design

**Figure 1:**
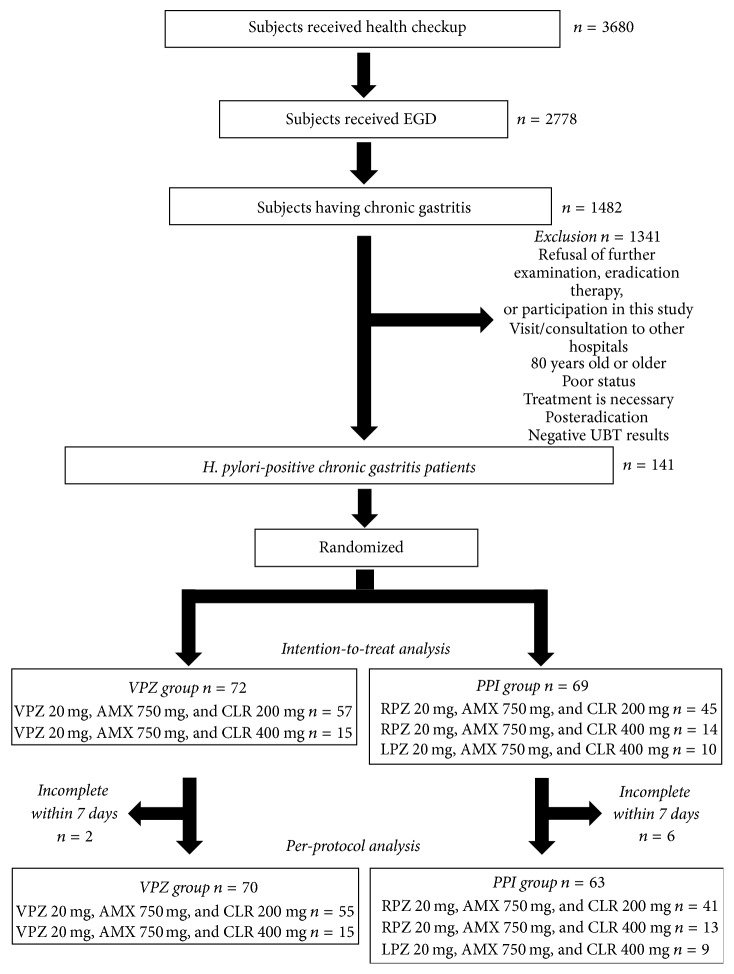
Flowchart of this study. The details were described in Methods section. EGD, esophagogastroduodenoscopy; UBT, urea breath test; VPZ, vonoprazan; AMX, amoxicillin; CLR, clarithromycin; PPI, proton pump inhibitor; RPZ, rabeprazole; LPZ, lansoprazole.

A phase IV randomized, single-blind, single-center, parallel-group comparative study was designed to assess the superiority of VPZ/AMX/CLR triple therapy to PPI/AMX/CLR therapy as a first-line* H. pylori* eradication. The enrollment criteria of the participants are shown in Figure 1. From April 2015 to February 2016, 3680 persons received health checkup in Nagano Red Cross Hospital. Among them, 2778 persons wished to undergo esophagogastroduodenoscopy as a survey of upper gastrointestinal tract and 1482 were endoscopically diagnosed as having chronic active gastritis. All chronic gastritis patients who visited our hospital for the treatment received ^13^C-urea breath test (UBT) using UBIT tablets (100 mg, Otsuka pharmaceutical, Tokyo, Japan) and Δ^13^C of more than 2.5‰ was diagnosed as having* H. pylori* infection. We excluded 1341 subjects because of one of the following reasons: refusal of further examination, eradication therapy, or participation in this study, visit/consultation to other hospitals, 80 years old or older, poor status, requiring immediate treatment, posteradication, or negative results for UBT. According to the report from Murakami et al. [21], we hypothesized that the eradication rate of VPZ/AMX/CLR combination therapy would be around 90%. Based on our experience and other reports [22, 23], a first eradication rate in PPI-based protocol was approximately 70%. In order to demonstrate a superiority of VPZ-based regimen for a first-line* H. pylori* eradication, we calculated a sample size for comparing 90% in VPZ-based regimen versus 70% in PPI-based one with a two-sided *α* of 0.05 and a power of 80% and required at least 62 participants in each arm. Consequently, 141 UBT-positive chronic gastritis patients were diagnosed as having* H. pylori* infection, approved to participate in this randomized control trial, and received eradication therapy. Randomization was performed according to the personal medical record number, that is, odd number to PPI group and even one to VPZ group.

### 2.3. Eradication Regimen

The patients in VPZ group received VPZ 20 mg, AMX 750 mg, and CLR 200 mg or 400 mg twice daily for 7 days. The patients in PPI group received rabeprazole (RPZ) 20 mg, AMX 750 mg, and CLR 200 mg or 400 mg twice daily for 7 days or LPZ 30 mg, AMX 750 mg, and CLR 400 mg twice daily for 7 day. Selection of RPZ or LPZ and CLR 200 mg or 400 mg was decided by each doctor.

### 2.4. Study Outcomes

The primary outcomes were* H. pylori* eradication rate and adverse event. Eradication success was evaluated using ^13^C-UBT at 8 weeks after completing the treatment and Δ^13^C of less than 2.5‰ was judged as successful eradication. The eradication rate was assessed by intention-to-treat (ITT) and per-protocol (PP) analysis. Adverse events were also evaluated at 8 weeks after finishing the treatment using a questionnaire.

### 2.5. Statistical Analyses

Statistical analyses were performed using the excel toukei (SSRI Co, Tokyo, Japan). Categorical and continuous variables were compared with *χ*^2^ test or Fisher's exact test (when the group contained 5 or less than 5 subjects) and Mann–Whitney *U* test, respectively. A* P* value of less than 0.05 was considered as statistically significant.

## 3. Results

### 3.1. Baseline Characteristics of the Participants

Among 141 participants, 72 and 69 subjects received VPZ-based and PPI-based treatment, respectively (Figure 1). There were no significant differences in clinical parameters of the patients, such as age, body mass index, frequency of male, smoker, drinker, comorbidities, serum levels of liver enzymes and creatinine, and glycohemoglobin values between the groups (Table 1). Two patients in the VPZ and 6 patients in the PPI group missed doses of the medications; therefore the completion rate was 97% (70/72) and 91% (63/69) in the VPZ and PPI group, respectively. No drop-off was observed due to serious adverse effects, treatment side effects, and withdrawal from study participation.

### 3.2. Eradication Rate

**Figure 2:**
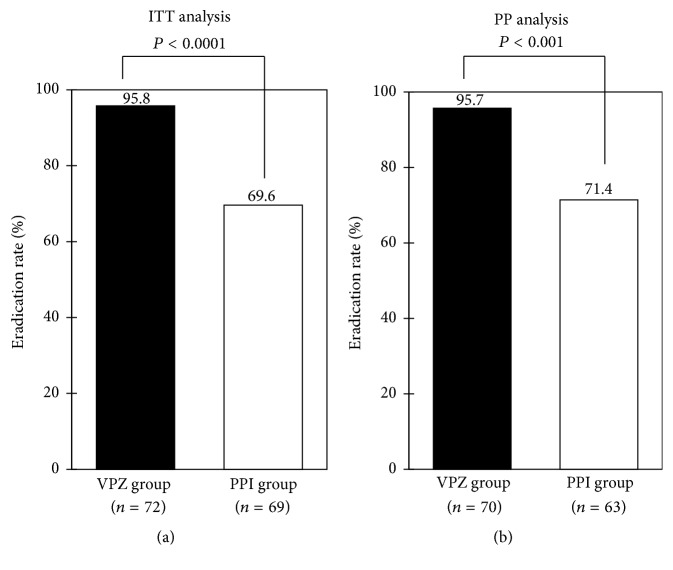
Comparison of eradication rates between vonoprazan- (VPZ-) based regimen and proton pump inhibitor- (PPI-) based regimen. Intention-to-treat (a) and per-protocol (b) analyses. Statistical analysis was performed by *χ*^2^ test. VPZ group, treatment with vonoprazan, amoxicillin, and clarithromycin; PPI group, treatment with rabeprazole or lansoprazole, amoxicillin, and clarithromycin.

**Figure 3:**
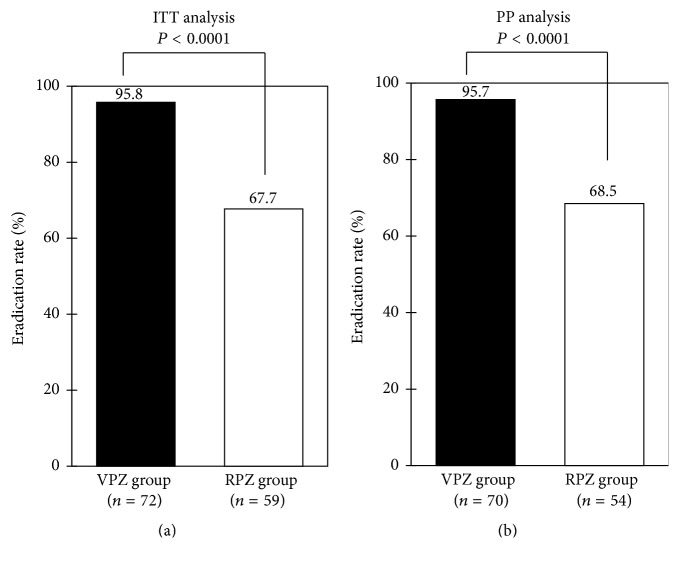
Comparison of eradication rates between vonoprazan- (VPZ-) based regimen and rabeprazole- (RPZ-) based regimen. Intention-to-treat (a) and per-protocol (b) analyses. Statistical analysis was performed by *χ*^2^ test. VPZ group, treatment with vonoprazan, amoxicillin, and clarithromycin; RPZ group, treatment with rabeprazole, amoxicillin, and clarithromycin.

The eradication rate was greater in VPZ-based therapy compared with PPI-based one by ITT analysis (95.8% versus 69.6%, *P* = 0.00003; 95% confidence interval [CI] 88.3–99.1 versus 57.3–80.1%) (Figure 2(a)), as well as PP analysis (95.7% versus 71.4%, *P* = 0.0002; 95% CI 88.0–99.1 versus 58.7–82.1%) (Figure 2(b)). When the eradication rate was compared between VPZ and RPZ, a widely prescribed PPI in Japan, it was significantly higher in the former regimen in both analyses (Figures 3(a) and 3(b)). Additionally, there were no significant differences in the eradication rate between 200 mg and 400 mg of CLR in VPZ group (94.7% versus 100%, *P* = 1.00) and RPZ group (75.6% versus 42.9%, *P* = 0.05), respectively, by ITT analysis, as well as PP analysis (94.5% versus 100%, *P* = 1.00 and 75.6% versus 46.2%, *P* = 0.10).

### 3.3. Adverse Events

Incidence of adverse events was not different between the groups (26.3% in VPZ group versus 37.7% in PPI group, *P* = 0.15). The common adverse events was loose stool, diarrhea, dysgeusia, and skin eruption (Table 2). Suppressed gastric acid secretion can raise circulating gastrin levels. However, serum gastrin concentrations at 8 weeks after the completion of treatment were similar between VPZ group (median 102 pg/mL, range 22–970) and PPI group (181 pg/mL, 23–1200) (*P* = 0.12).

## 4. Discussion

The current randomized controlled trial was designed to assess the efficacy and safety of VPZ-based triple therapy and verified its superiority to conventional PPI-based therapy as a first-line* H. pylori* eradication. The results in this prospective study were in agreement with the results of the recent phase III noninferior trial for* H. pylori*-positive patients with a history of gastric or duodenal ulcer. Overall, a new VPZ/AMX/CLR treatment can be recommended for naïve* H. pylori*-infected patients.

VPZ, a first-in-class potassium-competitive blocker, is structurally stable, activated even in gastric acid, and absorbed rapidly and reaches maximum plasma concentration at 1.5–2.0 hours after single oral administration [18–20]. It is highly concentrated in the acidic canaliculi of the gastric parietal cells, exerting an acid-suppressive effect for longer than 24 hours [18, 19]. Accordingly, VPZ raises the gastric pH levels above 4.0 as early as 4 hours after single administration and maintains them for more than 24 hours [18]. Indeed, the acid-inhibiting potency is approximately 350 times greater than LPZ [18]. Since the most suitable gastric pH for antibiotics is around 6, the strong, immediate, and persistent effect of VPZ likely contributes to higher eradication rate compared with PPI.

Another possible advantage of VPZ is that it is not metabolized through CYP2C19. PPI is mainly metabolized by CYP2C19 and its gene polymorphism can affect PPI elimination from the body. Furuta et al. reported that the patients carrying CYP2C19 genotype associated with rapid extensive PPI metabolizers had lower eradication success rate after PPI/AMX/CLR therapy compared with poor metabolizers [24]. The frequency of CYP2C19 genotype of rapid extensive metabolizers is reported to be approximately 30% of Asians and 80% of Caucasian, respectively [24]. Therefore, VPZ-based therapy is also useful for PPI rapid extensive metabolizers.

CLR-containing triple therapy for 7 to 14 days is a standard first-line regimen in Asia [14], but the eradication rate has fallen below 80% in many Asian countries [14], which is consistent with the results of the present study that the eradication rate of PPI/AMX/CLR therapy was as low as 70%. Reduced eradication rate of traditional PPI/AMX/CLR is presumably due to insufficient bacteria-killing effect of CLR. Indeed, CLR-insensitive rate was higher than 15% in several Asian countries, such as Japan, China, and India [14]. Greater eradication rate in VPZ/AMX/CLR compared with PPI/AMX/CLR suggests that VPZ enhances the antibacterial effect of CLR.

The limitation of this prospective trial was small number of the patients in a single medical center. Additionally, there were no significant differences in the incidence of adverse events between the both groups in the present study, but Suzuki et al. reported the incidence of skin rash was significantly higher in VPZ therapy [25]. Further large-scale multicenter studies are needed to confirm the efficacy and safety of VPZ-based treatment.

## 5. Conclusion

In conclusion, VPZ-based regimen is safe and more efficacious than PPI-based regimen as a first-line* H. pylori* eradication therapy. VPZ may be a key agent to improve* H. pylori* eradication rate and prevent gastric cancer.

## Figures and Tables

**Table 1:** Baseline characteristics of the participants.

	VPZ group (*n* = 72)	PPI group (*n* = 69)	*P* value VPZ versus PPI	RPZ group (*n* = 59)	*P* value VPZ versus RPZ
Age (yrs)	58 (32–80)	60 (36–77)	0.7196	61 (36–77)	0.9299
Male	41/72 (57%)	40/69 (58%)	0.9019	35/59 (59%)	0.7838
Body mass index (kg/m^2^)	22.4 (17.9–30.7)	22.6 (16.1–37.8)	0.4780	22.7 (16.1–37.8)	0.3382
Smoking	17/70 (24%)	19/67 (28%)	0.5883	15/57 (26%)	0.7932
Drinking	22/72 (31%)	22/67 (33%)	0.7727	18/57 (32%)	0.9007
Comorbidities					
Gastric ulcer	1/72 (1.4%)	1/69 (1.4%)	1.0000	1/59 (1.7%)	1.0000
Duodenal ulcer	2/72 (2.8%)	2/69 (2.9%)	1.0000	2/59 (3.4%)	1.0000
Diabetes	8/72 (11.1%)	2/69 (2.9%)	0.0976	2/59 (3.4%)	0.1838
Hypertension	14/72 (19.4%)	17/69 (24.6%)	0.4567	16/59 (27.1%)	0.2983
Hyperlipidemia	8/72 (11.1%)	14/69 (20.3%)	0.1333	14/59 (23.7%)	0.0546
AST (IU/L)	20 (12–37)^a^	21 (12–39)^b^	0.7424	21 (12–39)^c^	0.3131
ALT (IU/L)	17 (7–36)^a^	17 (8–68)^b^	0.4114	17 (9–68)^c^	0.1291
Creatinine (mg/dL)	0.78 (0.52–1.27)^a^	0.78 (0.47–1.21)^b^	0.6356	0.78 (0.47–1.21)^c^	0.3345
Hemoglobin A1c (%)	5.6 (5.0–7.4)^d^	5.6 (3.5–7.9)^e^	0.1300	5.6 (3.5–7.9)^f^	0.3101

Data are expressed as median (range) or number (percentage). Statistical analyses were conducted with the Mann–Whitney *U* test and chi-square test or Fisher's exact test. VPZ group, treatment with vonoprazan, amoxicillin, and clarithromycin; PPI group, treatment with rabeprazole or lansoprazole, amoxicillin, and clarithromycin; RPZ group, treatment with rabeprazole, amoxicillin, and clarithromycin; AST, aspartate aminotransferase; ALT, alanine aminotransferase. ^a^*n* = 71, ^b^*n* = 67, ^c^*n* = 57, ^d^*n* = 64, ^e^*n* = 63, and ^f^*n* = 54.

**Table 2:** Incidence of adverse events.

	VPZ group (*n* = 72)	PPI group (*n* = 69)	*P* value VPZ versus PPI	RPZ group (*n* = 59)	*P* value VPZ versus RPZ
Overall	19 (26.3%)	26 (37.7%)	0.1505	20 (33.9%)	0.3497
Loose stool	12 (16.6%)	12 (17.4%)	0.9089	9 (15.3%)	0.8265
Diarrhea	6 (8.3%)	10 (14.5%)	0.2490	8 (13.6%)	0.3354
Dysgeusia	3 (4.1%)	6 (8.7%)	0.3192	5 (8.5%)	0.4664
Skin eruption	1 (1.4%)	3 (4.3%)	0.3591	2 (3.4%)	0.5881
Abdominal bloating	1 (1.4%)	2 (2.9%)	0.6143	1 (1.7%)	1.0000
Epigastralgia	0 (0%)	2 (2.9%)	0.2377	2 (3.4%)	0.4504
Constipation	1 (1.4%)	1 (1.4%)	1.0000	1 (1.7%)	1.0000
Nausea	1 (1.4%)	1 (1.4%)	1.0000	1 (1.7%)	1.0000
Others	2 (2.8%)	4 (5.8%)	0.4348	3 (5.1%)	0.6571

Data are expressed as number (percentage). Statistical analyses were conducted with the chi-square test or Fisher's exact test. VPZ group, treatment with vonoprazan, amoxicillin, and clarithromycin; PPI group, treatment with rabeprazole or lansoprazole, amoxicillin, and clarithromycin; RPZ group, treatment with rabeprazole, amoxicillin, and clarithromycin.
